# Solution reaction design: electroaccepting and electrodonating powers of ions in solution

**DOI:** 10.1186/1556-276X-7-6

**Published:** 2012-01-05

**Authors:** Keyan Li, Min Li, Dongfeng Xue

**Affiliations:** 1 State Key Laboratory of Fine Chemicals, School of Chemical Engineering, Dalian University of Technology, Dalian 116024, People's Republic of China; 2 State Key Laboratory of Rare Earth Resource Utilization, Changchun Institute of Applied Chemistry, Chinese Academy of Sciences, Changchun 130022, People's Republic of China

**Keywords:** electroaccepting, electrodonating, solution phase, solvent effect

## Abstract

By considering a first-order variation in electroaccepting and electrodonating powers, *ω*^±^, induced by a change from gas to aqueous solution phase, the solvent effect on *ω*^± ^for charged ions is examined. The expression of electroaccepting and electrodonating powers in the solution phase, *ω*^±^_s_, is obtained through establishing the quantitative relationship between the change of the *ω*^± ^due to the solvation and the hydration free energy. It is shown that cations are poorer electron acceptors and anions are poorer electron donors in solution compared to those in gas phase. We have proven that the scaled aqueous electroaccepting power, *ω*^+^_s_, of cations can act as a good descriptor of the reduction reaction, which is expected to be applied in the design of solution reactions.

## Introduction

With the rapid development of functional materials, novel micro/nanostructures of the materials are highly demanded to obtain advanced properties, which can be achieved by the rational design of solution-phase chemical reactions [1-6]. Therefore, it is of significance to thoroughly understand the reactivity of chemical species and the mechanism of chemical reactions to further realize the solution reaction design. Among many chemical reactivity indices, one quantity of importance is the electrophilicity, *ω*, introduced by Parr and co-workers [7]. They defined *ω *as

(1) $$\omega =\mu^{2}/)2\eta$$

where *μ *is the chemical potential and *η *is the chemical hardness of an *N*-electron system with total energy, *E*, defined as *μ *= (∂^2 ^*E*/∂^2^*N*)_*v*(*r*) _and *η *= (∂^2 ^*E*/∂^2^*N*)_*v*(*r*)_. This index has been found to be helpful in analyzing the reactivity behaviors of a variety of compounds as well as the reaction mechanisms of diverse chemical processes [8,9]. As an important contribution to the *ω*, Gazauez et al. [10] argued that from a chemical perspective, it would make sense to differentiate the response of a system to the electron acceptance from the electron donation grounded on that the left and right derivatives of the total energy, *E*_DFT_(*N*), of an *N*-electron system with respect to the integer electron number, *N*, are different. By introducing an electron bath of nonzero chemical potential, *μ*_bath_, with which the chemical species can exchange electrons, they proposed electroaccepting [*ω*^+^] and electrodonating [*ω*^-^] powers as the following equation:

(2) $$\omega^{\pm }=\frac{{\left(\mu^{\pm }\right)}^{2}-{\left(\mu_{\text{bath}}\right)}^{2}}{2\eta^{\pm }}$$

where the chemical potential, *μ*^±^, and the chemical hardness, *η*^±^, were defined as

(3a) $$\mu^{+}=-\frac{\left(I+3A\right)}{4}\;\eta^{+}=\eta =\frac{I-A}{2}$$

(3b) $$\mu^{-}=-\frac{\left(3I+A\right)}{4}\;\eta^{-}=\eta =\frac{I-A}{2}$$

where *I *and *A *are the ionization potential and the electron affinity, respectively. A larger value of *ω*^+ ^corresponds to a larger capability of accepting charges, whereas a smaller value of *ω*^- ^implies a larger capability of donating charges.

Although some chemical phenomena have been rationalized by establishing the quantitative structure-reactivity relationships using these reactivity indices in the gas phase [11], the presence of solvent is bound to affect the reactivity behaviors of chemical substances. Therefore, studies on the reactivity indices such as *ω *and *ω*^± ^in solution are quite necessary to reveal the accurate reactivity of chemical species in solution and further predict and design the solution phase reactions [12]. While several theoretical calculations about the solvent effect on the *ω *for various chemical species have been performed [12-14], the solvent effect on the *ω*^± ^which are regarded as better descriptors of the donor-acceptor type interactions [10] has not received much attention to date. In this work, the solvent effect on the *ω*^± ^is estimated by establishing a linear relationship between the change of the *ω*^± ^due to the solvation and the hydration free energy, Δ*G*_hyd_. The values of aqueous electroaccepting power, *ω*^+^_s_, of 39 metal cations are quantitatively calculated, which are proven to be appropriate descriptors for the reduction reactions.

## Method

In a previous study, Perez et al. [14] examined the solvent effect on the electrophilicity index, *ω*, by introducing a first-order finite variation in the *ω *due to the solvation

(4) $$\Delta \omega_{\text{g}\to \text{s}}=\left(\frac{\mu_{\text{g}}}{\eta_{\text{g}}}\right)\Delta \mu_{\text{g}\to \text{s}}-\frac{1}{2}{\left(\frac{\mu_{\text{g}}}{\eta_{\text{g}}}\right)}^{2}\Delta \eta_{\text{g}\to \text{s}}=\Delta \omega_{1,\text{g}\to \text{s}}+\Delta \omega_{2,\text{g}\to \text{s}}$$

where Δ*μ*_g→s _and Δ*η*_g→s _are the variations in *μ *and *η *from the gas to solution phase, respectively.

They rearranged the first contribution as

(5) $$\Delta \omega_{1,\text{g}\to \text{s}}=\left(\frac{\mu_{\text{g}}}{\eta_{\text{g}}}\right)\Delta \mu_{\text{g}\to \text{s}}={\left[\frac{\Delta E_{\text{g}}}{\Delta N_{\text{g}}}\right]}_{\upsilon \left(r\right)}\left[\frac{\Delta N_{\text{g}}}{\Delta \mu_{\text{g}}}\right]\Delta \mu_{\text{g}\to \text{s}}≅\Delta E_{\text{ins}}=2\Delta G_{\text{solv}}$$

where Δ*E*_ins _is the insertion energy of the solute going into the solvent which is suggested as twice the solvation energy.

The second contribution in Equation 4 is rewritten as

(6) $$\begin{array}{ccc} \Delta \omega_{2,\text{g}\to \text{s}} & =-\frac{1}{2}{\left(\frac{\mu_{\text{g}}}{\eta_{\text{g}}}\right)}^{2}\Delta \eta_{\text{g}\to \text{s}}=-\frac{1}{2}\left(\frac{\mu_{\text{g}}}{\eta_{\text{g}}}\right)\left(\frac{\mu_{\text{g}}}{\eta_{\text{g}}}\frac{\Delta \mu }{\Delta N}\right)=-\frac{1}{2}\left(\frac{\mu_{\text{g}}}{\eta_{\text{g}}\Delta N}\right)\left(\frac{\mu_{\text{g}}}{\eta_{\text{g}}}\Delta \mu \right). & \\ & =-\frac{1}{2}\left(\frac{\mu_{\text{g}}}{\eta_{\text{g}}\Delta N}\right)\Delta E_{\text{ins}}\;=\frac{\Delta N_{\text{g},\max}}{\Delta N}\Delta G_{\text{solv}} \end{array}$$

Finally, they deduced the expression of Δ*ω*_g→s_

(7) $$\Delta \omega_{\text{g}\to \text{s}}=\Delta \omega_{1,\text{g}\to \text{s}}+\Delta \omega_{2,\text{g}\to \text{s}}=\left(2+\frac{\Delta N_{\text{g},\max}}{\Delta N}\right)\Delta G_{\text{solv}}=\gamma \Delta G_{\text{solv}}$$

where Δ*ω*_g→s _showed a linear dependence on the solvation energy, Δ*G*_solv_, with a regression slope, *γ*. They used 18 well-known electrophilic ligands including hard electrophiles such as Li^+ ^and Na^+ ^to test this linear correlation and obtained good results (*R *= 0.9925, *γ *= 1.00765 at B3LYP/6-311G**and *R *= 0.9918, *γ *= 0.96843 at HF/6-311G**levels of theory).

Herein, we reconstruct the second contribution in Equation 4 which will directly lead to a quantitative expression for Δ*ω*_g→s _with a definite slope value, *γ*.

(8) $$\begin{array}{ccc} \Delta \omega_{2,\text{g}\to \text{s}} & =-\frac{1}{2}{\left(\frac{\mu_{\text{g}}}{\eta_{\text{g}}}\right)}^{2}\Delta \eta_{\text{g}\to \text{s}}=-\frac{1}{2}{\left(\Delta N_{\text{g},\max}\right)}^{2}\Delta \eta_{\text{g}\to \text{s}} & \\ & =-\frac{1}{2}{\left(\Delta N_{\text{g},\max}\right)}^{2}\left[\left(\frac{\Delta \mu_{\text{s}}}{\Delta N_{\text{s},\max}}\right)-\left(\frac{\Delta \mu_{\text{g}}}{\Delta N_{\text{g},\max}}\right)\right]≅-\frac{1}{2}{\left(\Delta N_{\text{g},\max}\right)}^{2}\frac{\Delta \mu_{\text{s}}-\Delta \mu_{\text{g}}}{\Delta N_{\text{g},\max}}\\ & =-\frac{1}{2}{\left(\Delta N_{\text{g},\max}\right)}^{2}\frac{\left(0-\mu_{\text{s}}\right)-\left(0-\mu_{\text{g}}\right)}{\Delta N_{\text{g},\max}}=-\frac{1}{2}{\left(\Delta N_{\text{g},\max}\right)}^{2}\frac{\mu_{\text{g}}-\mu_{\text{s}}}{\Delta N_{\text{g},\max}}\\ & =-\frac{1}{2}\Delta N_{\text{g},\max}\left(-\Delta \mu_{\text{g}\to \text{s}}\right)=-\frac{1}{2}\left(-\frac{\mu_{\text{g}}}{\eta_{\text{g}}}\right)\left(-\Delta \mu_{\text{g}\to \text{s}}\right)=-\frac{1}{2}\Delta \omega_{1,\text{g}\to \text{s}}=-\Delta G_{\text{solv}} \end{array}$$

Substitution of Equations 5 and 8 into Equation 4 leads to the expression of Δ*ω*_g→s_.

(9) $$\Delta \omega_{\text{g}\to \text{s}}=\Delta \omega_{1,\text{g}\to \text{s}}+\Delta \omega_{2,\text{g}\to \text{s}}=2\Delta G_{\text{solv}}-\Delta G_{\text{solv}}=\Delta G_{\text{solv}}$$

Therefore, the global electrophilicity, *ω*_s_, in solution can be calculated by

(10) $$\omega_{\text{s}}=\omega_{\text{g}}+\Delta \omega_{\text{g}\to \text{s}}=\omega_{\text{g}}+\Delta G_{\text{solv}}.$$

It should be noted that one key assumption in our approach is Δ*N*_s, max _≈ Δ*N*_g, max _which could be justified by the data of Table 1 in Perez's work [14]. Our result, *γ *= 1, has turned out to be fairly consistent with Perez's regression value, i.e., *γ *= 1.00765 and *γ *= 0.96843, which thus approve the reasonableness of our approach to dealing with Δ*ω*_2, g→s_.

**Table 1:** Calculated electroaccepting power, *ω*^+^_s_, in aqueous solution and the absolute reduction potential, *E*°_abs_

*M*^z+^	*μ*_bath_^a^	*μ*^+ b^	*η*^+ b^	Δ*N*^+^_g, max_	Δ*G*_hyd_	Δ*ω*^+^	*ω*^+^_s_	z*E*°_abs_^c^
Li^+^	-4.044	-22.954	35.124	0.538	-481	-2.053	5.214	1.223
Na^+^	-3.854	-15.677	21.075	0.561	-375	-1.466	4.013	1.550
K^+^	-3.255	-11.163	13.645	0.580	-304	-1.116	3.062	1.339
Rb^+^	-3.133	-9.953	11.551	0.590	-281	-0.998	2.865	1.339
Cs^+^	-2.920	-9.195	10.603	0.592	-258	-0.912	2.673	1.340
Ag^+^	-5.682	-11.055	6.957	0.772	-440	-1.108	5.354	5.062
Cu^+^	-5.795	-10.867	6.282	0.807	-535	-1.294	5.433	4.783
Tl^+^	-4.581	-9.688	7.160	0.713	-310	-0.847	4.242	3.923
In^+^	-4.340	-9.057	6.542	0.721	-296	-0.799	4.032	4.123
Be^2+^	-6.992	-52.133	67.843	0.665	-2404	-10.787	8.883	4.546
Mg^2+^	-5.735	-31.313	32.554	0.786	-1838	-7.780	6.774	3.814
Ca^2+^	-4.585	-21.632	19.521	0.873	-1515	-6.187	5.260	2.846
Sr^2+^	-4.271	-18.995	15.930	0.924	-1386	-5.567	5.185	2.746
Ba^2+^	-3.909	-16.378	12.748	0.978	-1258	-4.963	4.958	2.686
V^2+^	-5.060	-18.291	7.347	1.801	-1825	-6.841	14.187	6.266
Cr^2+^	-5.075	-20.105	7.237	2.077	-1860	-7.206	18.940	6.726
Mn^2+^	-5.576	-20.147	9.014	1.617	-1770	-6.634	14.157	6.186
Fe^2+^	-5.927	-19.804	7.232	1.919	-1848	-6.711	17.976	7.646
Co^2+^	-5.911	-21.188	8.208	1.861	-1922	-7.181	18.037	7.972
Ni^2+^	-5.730	-22.424	8.511	1.962	-1998	-7.708	19.906	8.012
Cu^2+^	-5.795	-24.429	8.275	2.252	-2016	-7.969	26.064	9.206
Zn^2+^	-7.046	-23.405	10.879	1.504	-1963	-7.110	15.784	7.001
Cd^2+^	-6.745	-22.051	10.286	1.488	-1736	-6.244	15.180	7.720
Hg^2+^	-7.828	-22.618	7.722	1.915	-1766	-5.984	23.174	10.234
Sn^2+^	-5.508	-18.600	7.936	1.650	-1496	-5.457	14.429	8.251
Pb^2+^	-5.562	-19.258	8.453	1.620	-1434	-5.285	14.824	8.274
Pd^2+^	-6.253	-22.805	6.750	2.452	-1920	-7.222	28.406	10.356
Sm^2+^	-4.233	-14.153	6.165	1.609	-1375	-4.994	9.797	3.186
Eu^2+^	-4.253	-14.668	6.835	1.524	-1391	-5.118	9.296	2.926
Yb^2+^	-4.691	-15.395	6.437	1.663	-1510	-5.441	11.259	2.926
Al^3+^	-4.489	-51.334	45.772	1.023	-4531	-21.427	7.139	7.761
Ga^3+^	-4.499	-39.033	16.645	2.075	-4521	-20.728	24.430	11.202
In^3+^	-4.340	-34.523	12.985	2.324	-3989	-18.073	27.093	11.775
Sc^3+^	-4.921	-36.940	24.366	1.314	-3801	-17.073	10.431	6.699
Y^3+^	-4.663	-30.539	20.039	1.291	-3457	-15.179	7.550	5.679
La^3+^	-4.183	-26.870	15.386	1.475	-3155	-13.805	9.090	5.649
Fe^3+^	-5.927	-36.689	12.074	2.548	-4271	-18.557	35.731	12.669
Co^3+^	-5.911	-37.950	8.900	3.600	-4503	-19.701	59.247	14.148
Au^3+^	-6.919	-41.575	8.350	4.150	-4416	-19.075	81.561	17.349

^a^${\mu^{+}}_{\text{bath}}={\mu^{-}}_{\text{atom}}=-\left(3I_{\text{M}}+A_{\text{M}}\right)/)4≅-3I_{\text{M}}/)4$ because of the neglectable values of electron affinities, *A*_M_, for atoms. ^b ^*μ^+ ^*and *η*^+ ^are obtained from Equation 3a. ^c ^The product of the charge number, *z*, and the absolute reduction potential, *E*°_abs_. The values of *E*°_abs _are calculated form Equation 18 where the absolute standard hydrogen electrode potential, *E*°_SHE = 0_, is 4.263 eV according to Marcus [21].

Further, we try to extend our approach to examine the solvent effect on the *ω*^±^. For the charged ions, we suppose that the chemical potential, *μ*^±^_bath_, of the electron bath equals that of the parent atoms of ions since the charged ions become neutral atoms after accepting or donating the maximum amount of electrons. In addition, as the solvent only has little effect on the chemical potential, *μ*, of the neutral species [12,13,15], there exists a relationship as *μ*^±^_bath _= *μ*_s, atom _≈ *μ*_g, atom_. The ion exchanges electrons from the bath to the point that its chemical potential, *μ*^±^, equals the value *μ*^±^_bath _with the maximum amount of electron flow:

(11) $$\Delta {N^{\pm }}_{\max}=\left({\mu^{\pm }}_{\text{bath}}-\mu^{\pm }\right)/)\eta^{\pm }.$$

The first-order variation in the *ω*^± ^leads to the following equation:

(12) $$\Delta {\omega^{\pm }}_{\text{g}\to \text{s}}=\left(\frac{{\mu^{\pm }}_{\text{g}}}{{\eta^{\pm }}_{\text{g}}}\right)\Delta {\mu^{\pm }}_{\text{g}\to \text{s}}-\frac{1}{2}\frac{{\left({\mu^{\pm }}_{\text{g}}\right)}^{2}-{\left({\mu^{\pm }}_{\text{bath}}\right)}^{2}}{{\left({\eta^{\pm }}_{\text{g}}\right)}^{2}}\Delta {\eta^{\pm }}_{\text{g}\to \text{s}}=\Delta {\omega^{\pm }}_{1,\text{g}\to \text{s}}+\Delta {\omega^{\pm }}_{2,\text{g}\to \text{s}}.$$

The first part of Equation 12 in terms of the variation in *μ*^± ^is given by

(13) $$\Delta {\omega^{\pm }}_{1,\text{g}\to \text{s}}=\left(\frac{{\mu^{\pm }}_{\text{g}}}{{\eta^{\pm }}_{\text{g}}}\right)\Delta {\mu^{\pm }}_{\text{g}\to \text{s}}={\left[\frac{\Delta E_{\text{g}}}{\Delta {N^{\pm }}_{\text{g}}}\right]}_{\upsilon \left(r\right)}\left[\frac{\Delta {N^{\pm }}_{\text{g}}}{\Delta {\mu^{\pm }}_{\text{g}}}\right]\Delta {\mu^{\pm }}_{\text{g}\to \text{s}}≅\Delta G_{\text{hyd}}$$

where the energy change Δ*ω*^±^_1,g→s _due to the variation of the chemical potential from the gas to solution phase can be represented by Δ*G*_hyd _[16,17].

The second part of Equation 12 in terms of the variation in *η*^± ^is given by

(14) $$\begin{array}{ccc} \Delta {\omega^{\pm }}_{2,\text{g}\to \text{s}} & =-\frac{1}{2}\frac{{\left({\mu^{\pm }}_{\text{g}}\right)}^{2}-{\left({\mu^{\pm }}_{\text{bath}}\right)}^{2}}{{\left({\eta^{\pm }}_{\text{g}}\right)}^{2}}\Delta {\eta^{\pm }}_{\text{g}\to \text{s}}=-\frac{1}{2}\frac{{\left({\mu^{\pm }}_{\text{g}}\right)}^{2}-{\left({\mu^{\pm }}_{\text{bath}}\right)}^{2}}{{\left({\eta^{\pm }}_{\text{g}}\right)}^{2}}\Delta \left(\frac{\Delta \mu^{\pm }}{\Delta N^{\pm }}\right) & \\ & =-\frac{1}{2}\frac{{\left({\mu^{\pm }}_{\text{g}}\right)}^{2}-{\left({\mu^{\pm }}_{\text{bath}}\right)}^{2}}{{\left({\eta^{\pm }}_{\text{g}}\right)}^{2}}\left[\left(\frac{\Delta {\mu^{\pm }}_{\text{s}}}{\Delta N_{\text{s},\max}}\right)-\left(\frac{\Delta {\mu^{\pm }}_{\text{g}}}{\Delta N_{\text{g},\max}}\right)\right]\\ & ≅-\frac{1}{2}\frac{{\left({\mu^{\pm }}_{\text{g}}\right)}^{2}-{\left({\mu^{\pm }}_{\text{bath}}\right)}^{2}}{{\left({\eta^{\pm }}_{\text{g}}\right)}^{2}}\frac{\left({\mu^{\pm }}_{\text{bath}}-{\mu^{\pm }}_{\text{s}}\right)-\left({\mu^{\pm }}_{\text{bath}}-{\mu^{\pm }}_{\text{g}}\right)}{\Delta N_{\text{g},\max}}\\ & =\frac{1}{2}\frac{{\left({\mu^{\pm }}_{\text{g}}\right)}^{2}-{\left({\mu^{\pm }}_{\text{bath}}\right)}^{2}}{{\left({\eta^{\pm }}_{\text{g}}\right)}^{2}}\frac{{\eta^{\pm }}_{\text{g}}}{\Delta {\mu^{\pm }}_{\text{g}}}\Delta {\mu^{\pm }}_{\text{g}\to \text{s}}\\ & =-\frac{1}{2}\frac{{\mu^{\pm }}_{\text{bath}}+{\mu^{\pm }}_{\text{g}}}{{\eta^{\pm }}_{\text{g}}}\Delta {\mu^{\pm }}_{\text{g}\to \text{s}}=-\frac{1}{2}\frac{{\mu^{\pm }}_{\text{bath}}+{\mu^{\pm }}_{\text{g}}}{{\mu^{\pm }}_{\text{g}}}\frac{{\mu^{\pm }}_{\text{g}}}{{\eta^{\pm }}_{\text{g}}}\Delta {\mu^{\pm }}_{\text{g}\to \text{s}}\\ & =-\frac{1}{2}\frac{{\mu^{\pm }}_{\text{bath}}+{\mu^{\pm }}_{\text{g}}}{{\mu^{\pm }}_{\text{g}}}\Delta {\omega^{\pm }}_{1,\text{g}\to \text{s}} \end{array}$$

Combining Equation 13 with Equation 14 yields

(15) $$\begin{array}{ccc} \Delta {\omega^{\pm }}_{\text{g}\to \text{s}} & =\Delta {\omega^{\pm }}_{1,\text{g}\to \text{s}}+\Delta {\omega^{\pm }}_{2,\text{g}\to \text{s}}=\;\left(\text{1}-\frac{1}{2}\frac{{\mu^{\pm }}_{\text{bath}}+{\mu^{\pm }}_{\text{g}}}{{\mu^{\pm }}_{\text{g}}}\right)\Delta {\omega^{\pm }}_{1,\text{g}\to \text{s}} & \\ & =\left(\text{1}-\frac{1}{2}\frac{{\mu^{\pm }}_{\text{bath}}+{\mu^{\pm }}_{\text{g}}}{{\mu^{\pm }}_{\text{g}}}\right)\Delta G_{\text{hyd}}. \end{array}$$

Therefore, the electroaccepting and electrodonating powers in solution, *ω*^±^_s_, can be calculated by

(16) $${\omega^{\pm }}_{\text{s}}={\omega^{\pm }}_{\text{g}}+\Delta {\omega^{\pm }}_{\text{g}\to \text{s}}=\frac{{\left({\mu^{\pm }}_{\text{g}}\right)}^{2}-{\left(\mu_{\text{bath}}\right)}^{2}}{2{\eta^{\pm }}_{\text{g}}}+\left(\text{1}-\frac{1}{2}\frac{{\mu^{\pm }}_{\text{bath}}+{\mu^{\pm }}_{\text{g}}}{{\mu^{\pm }}_{\text{g}}}\right)\Delta G_{\text{hyd}}.$$

## Results and discussion

According to Pearson's viewpoint that cations are electron acceptors and anions are electron donors [16], we pay attention to the *ω*^+^_s _for cations and *ω*^-^_s _for anions. By using Equation 16, the *ω*^+^_s _values for 39 metal cations with charges from +1e to +3e are calculated and summarized in Table 1. From Table 1, we find that the solvation weakens the capacity of cations to accept electrons due to the negative values of Δ*ω*^+^, in agreement with the previous conclusions [12-16]. Unfortunately, it is impossible to quantitatively calculate the *ω*^-^_s _values for anions so far due to the absence of experimental electron affinities needed in Equation 3b. Herein, these values can be qualitatively estimated:

(17) $$\begin{array}{ccc} {\omega^{-}}_{\text{s}} & ={\omega^{-}}_{\text{g}}+\Delta {\omega^{-}}_{\text{g}\to \text{s}}={\omega^{-}}_{\text{g}}+\left(\text{1}-\frac{1}{2}\frac{{\mu^{-}}_{\text{bath}}+{\mu^{-}}_{\text{g}}}{{\mu^{-}}_{\text{g}}}\right)\Delta G_{\text{hyd}} & \\ & >{\omega^{-}}_{\text{g}}+\left(\text{1}-\frac{1}{2}\frac{{\mu^{-}}_{\text{g}}+{\mu^{-}}_{\text{g}}}{{\mu^{-}}_{\text{g}}}\right)\Delta G_{\text{hyd}}\;>{\omega^{-}}_{\text{g}}. \end{array}$$

Since a larger value of *ω*^-^_s _implies a smaller capability of donating charges, we can conclude from Equation 17 that the solvation also weakens the capacity of anions to donate electrons, which agrees with the general viewpoints [12-16].

**Figure 1:**
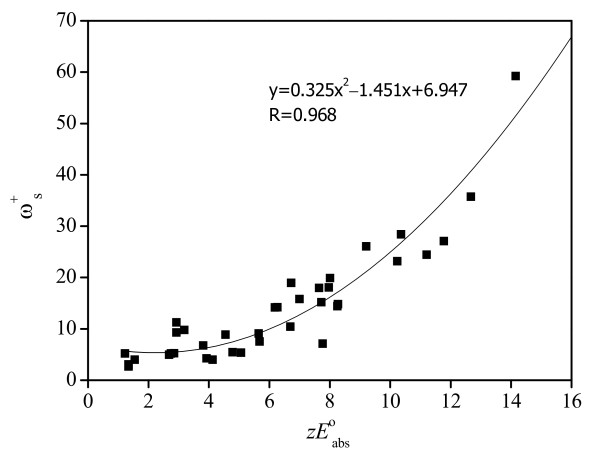
**Plot of the *ω*^+^_s _versus *zE*°_abs_**.

Many liquid-phase chemical reactions involve the electron-transfer steps, and a key thermodynamic variable that describes the tendency of chemical species in solution to gain or lose electrons is the redox potential. The quantum-chemical computation approach to electrochemistry has become available very recently [18]. However, the estimation of redox potential by the quantum-chemical calculations is a great challenge due to the complexity of the processes involved in a typical electrochemical reaction [19]. For example, the complicated diffusion and adsorption processes on the electrode surface which should be necessarily taken into account in the quantum-chemical modeling of the reduction-oxidation reaction lead to the considerable system size and thus require strong computing power. Therefore, previous studies mainly focus on the one-electron reduction reactions between different oxidation states of transition metals to avoid modeling of an electrode-solution boundary [18-20]. In this work, we try to use the *ω*^+^_s _to describe the many-electron reduction reaction including both main- and sub-group metal cations. According to the reaction formula *M*^*Z*+ ^(aq) + *z*/2H_2 _(g) = *M *(aq) + *z*H^+ ^(aq), the absolute reduction potential, *E*°_abs_, can be calculated by

(18) $$E_{\text{abs}}^{\circ }=E_{\text{SHE}=0}^{\circ }+E_{\text{abs}}^{\circ }\left(\text{SHE}\right)$$

where *E*°_abs_(SHE) is the conventional reduction potential and *E*°_SHE = 0 _is the absolute standard hydrogen electrode potential. Note that the *ω*^+^_s _is the energy lowing associated with a maximum amount of electron flow between two species; it is reasonable to establish a correlation between z*E*°_abs _and *ω*^+^_s_. A good relationship shown in Figure 1 approves that our *ω*^+^_s _can act as an appropriate descriptor of the many-electron energy change. Moreover, this method is more simple and convenient compared to the quantum-chemical approach to the estimation of the *E*°_abs_.

Except for the reduction reaction, the *ω*^±^_s _can also be expected to qualitatively and quantitatively predict other properties of ions in connection with ligand binding, hydrolysis processes, and stability of coordination compounds, etc. In addition, compilation of experimental data on solvation energies in nonaqueous solutions will make it possible to evaluate the corresponding electroaccepting and electrodonating powers, *ω*^±^, which will undoubtedly lead to the deeper understanding of the chemical reactivity of ions in these media.

## Conclusions

By reconstructing a first-order variation of the *ω *due to the solvation, the linear relationship between the change in the *ω *and the solvation energy is reproduced, which suggests that our method is theoretically reasonable. The solvent effect on the electroaccepting and electrodonating powers, *ω*^±^, for charged ions is examined, and a definite quantitative expression for the aqueous *ω*^±^_s _is established. It is found that the solvation weakens the capability of both electron-accepting power of cations and electron-donating power of anions. A good relationship between the *ω^+^*_s _and *E*°_abs _shows the validity of the electroaccepting powers in determining the chemical reactivity of the ions in aqueous solution. It is expected that our *ω*^±^_s _will be helpful to achieve a better understanding of chemical properties of ions in solution and further be used in many aspects of solution chemistry such as the design of solution-phase reactions according to these indices.

## Competing interests

The authors declare that they have no competing interests.

## Authors' contributions

KL participated in the design and coordination of the study and corrected the manuscript. ML assisted in the calculation of the data and prepared the manuscript initially. DX conceived the study, supervised, and corrected the manuscript. All authors read and approved the final manuscript.
